# Application of human platelet lysate in chondrocyte expansion promotes chondrogenic phenotype and slows senescence progression via BMP–TAK1–p38 pathway

**DOI:** 10.1038/s41598-023-48544-0

**Published:** 2023-11-30

**Authors:** Narong Chitchongyingcharoen, Tulyapruek Tawonsawatruk, Jitrada Phetfong, Wrattya Aroontanee, Aungkura Supokawej

**Affiliations:** 1 Department of Clinical Microscopy, Faculty of Medical Technology, Mahidol University, 999 Phutthamonthon Sai 4, Salaya, Phutthamonthon, Nakhon Pathom, 73170 Thailand; 2 Department of Orthopedics, Faculty of Medicine, Ramathibodi Hospital, Mahidol University, Bangkok, Thailand

**Keywords:** Cell biology, Molecular biology

## Abstract

Osteoarthritis (OA) is one of the most common musculoskeletal degenerative. OA treatments are aiming to slow down disease progression; however, lack of cartilage regeneration efficacy. Autologous chondrocyte implantation (ACI) is a promising cartilage-regeneration strategy that uses human articular chondrocytes (HACs) as cellular materials. However, the unreadiness of HACs from prolonged expansion, cellular senescence, and chondrogenic dedifferentiation occurred during conventional expansion, thus, minimizing the clinical efficacy of ACI. We aimed to examine the effects of a human platelet lysate (HPL) as an alternative human-derived HAC medium supplement to overcome the limitations of conventional expansion, and to explain the mechanism underlying the effects of HPL. During passages 2–4 (P2-P4), HPL significantly increased HAC proliferation capacities and upregulated chondrogenic markers. Simultaneously, HPL significantly reduced HAC senescence compared with conventional condition. HACs treated with LDN193189 exhibited a reduction in proliferation capacity and chondrogenic marker expression, whereas the HAC senescence increased slightly. These findings indicated involvement of BMP-2 signaling transduction in the growth-assistive, anti-senescent, and chondrogenic-inductive properties of HPL, which demonstrated its beneficial effects for application as HAC medium supplement to overcome current expansion limitations. Finally, our findings support the roles of platelets in platelet-rich plasma as a promising treatment for patients with OA.

## Introduction

In the upcoming decade, the global aging of societies will impact all countries worldwide. The demographic shift into an aging society is strongly associated with several age-related degenerative diseases. Conditions of cartilage degeneration, such as osteoarthritis (OA), are among the most common musculoskeletal degenerative diseases and affect the mobility and quality of life of patients. The common treatments available for OA are based on supportive strategies aimed at slowing down disease progression; however, these strategies do not afford hyaline cartilage regeneration^1–3^. OA progression starts with an asymptomatic phase, followed by an irreversible progression, which necessitates total knee replacement. Aiming for cartilage regeneration, several techniques and biologics for injection were studied based on the usage of products such as hyaluronic acid^4,5^, platelet-rich plasma (PRP)^6,7^, mesenchymal stem cells (MSCs), and human articular chondrocytes (HACs)^8–12^. Hyaluronic acid and PRP injection yielded transient pain relief; however, their effects on cartilage regeneration remain controversial^13–17^. In contrast, human MSCs and HACs, as cellular materials, were able to improve hyaline cartilage regeneration in patients^18–21^.

Autologous chondrocyte implantation (ACI), a cartilage-regeneration strategy that uses HACs as cellular materials, has been established and developed as a promising strategy for damaged cartilage tissue regeneration. It exhibited a success rate of approximately 85% and afforded efficient hyaline cartilage regeneration and pain reduction; moreover, it increased the movement capabilities of patients. However, several factors minimized clinical outcomes of ACI, including the age of the patients, area of defects, and quality of the *in-vitro*-expanded HACs^22^. Treatment of patients with OA with unready HACs was associated with a poor clinical outcome caused by fibrocartilage regeneration^23,24^. The difficulties of in vitro HAC expansion have been reported as follows: it is a time-consuming process that may lead to HAC senescence and the gradual loss of chondrogenic phenotype by acquisition of fibroblastoid characteristics via a process known as the dedifferentiation phenomenon^25,26^.

Dedifferentiated HACs exhibit an altered morphology and intracellular cytoskeletal architecture to adapt to the plastic cell-culture surface during in vitro HAC expansion. Moreover, the master regulatory factor of the chondrogenic phenotype, SOX9, is downregulated in these conditions, which hinders the production of type II collagen which is the main components of regenerated hyaline cartilage^27^. To overcome HAC dedifferentiation, several studies have reported alternative strategies for in vitro HAC expansion, such as adaptation of HACs to the culture environment into 3-dimensional expansion, and supplementation with specific growth factors, such as bone morphogenetic protein 2 (BMP-2), BMP-7, and fibroblast growth factor 2 (FGF-2), which can upregulate HAC chondrogenic markers, compared with fetal bovine serum (FBS), current HAC growth medium supplements^28–33^.

Seeking to enrich BMP-2-source supplements, the human platelet lysate (HPL) is a well-known potent human-derived blood product composed of BMP-2, BMP-7, and FGF-2 as potent chondrogenic inducers that reside in alpha granules of human platelet cells. Furthermore, HPL has been reported to accelerate the proliferative capacities of numerous human cell types, such as bone-marrow-derived MSCs (BMSCs), umbilical-cord-derived MSCs (UCMSCs), gingival stem cells, corneal endothelial cells, and HACs^34–36^. HACs are classified as advanced therapy medicinal products for clinical application, concerning good manufacturing practices. Alternative xenogenic (animal-derived) blood products, such as FBS, must be avoided to ensure the safety of patients. In turn, the identification of potent human-derived blood products for in vitro HAC expansion is essential to maximize the standard and quality of in vitro laboratory-expanded HACs. In addition to the characteristics mentioned above, HPL originating from pooled leukocyte poor platelet concentrates (LPPC) units was able to minimize human biological variation, leading to a stable growth factor content among the lots of HACs for medium preparation. To address the increasing trend of clinical-grade HAC expansion, HPL might be a promising alternative growth medium supplement to substitute the current use of FBS, all of which are critical key factors that maximize HAC quality and the clinical outcome of ACI treatment^37^.

The study aims to determine the effects of HPL in the culture medium on HAC growth and phenotypic changes. The senescence characteristics of HACs during in vitro expansion exhibited progression, which was alleviated by culturing in the presence of HPL. The amelioration of the dedifferentiation and senescence characteristics of HACs cultured with HPL proceeded via the BMP signaling cascade. Using a BMPRI receptor inhibitor (LDN193189), HPL, BMP, TAK1, and p38, signaling transduction interference was assessed to determine how HPL affects HAC properties. These findings support the need of using HPL in HAC culture for ACI purposes and highlight the role of using platelets in the treatment of OA.

## Results

### HPL maintained normal HAC karyotypes and a low-level PDT while increasing HAC proliferative capacities during four passages of in vitro expansion

**Figure 1:**
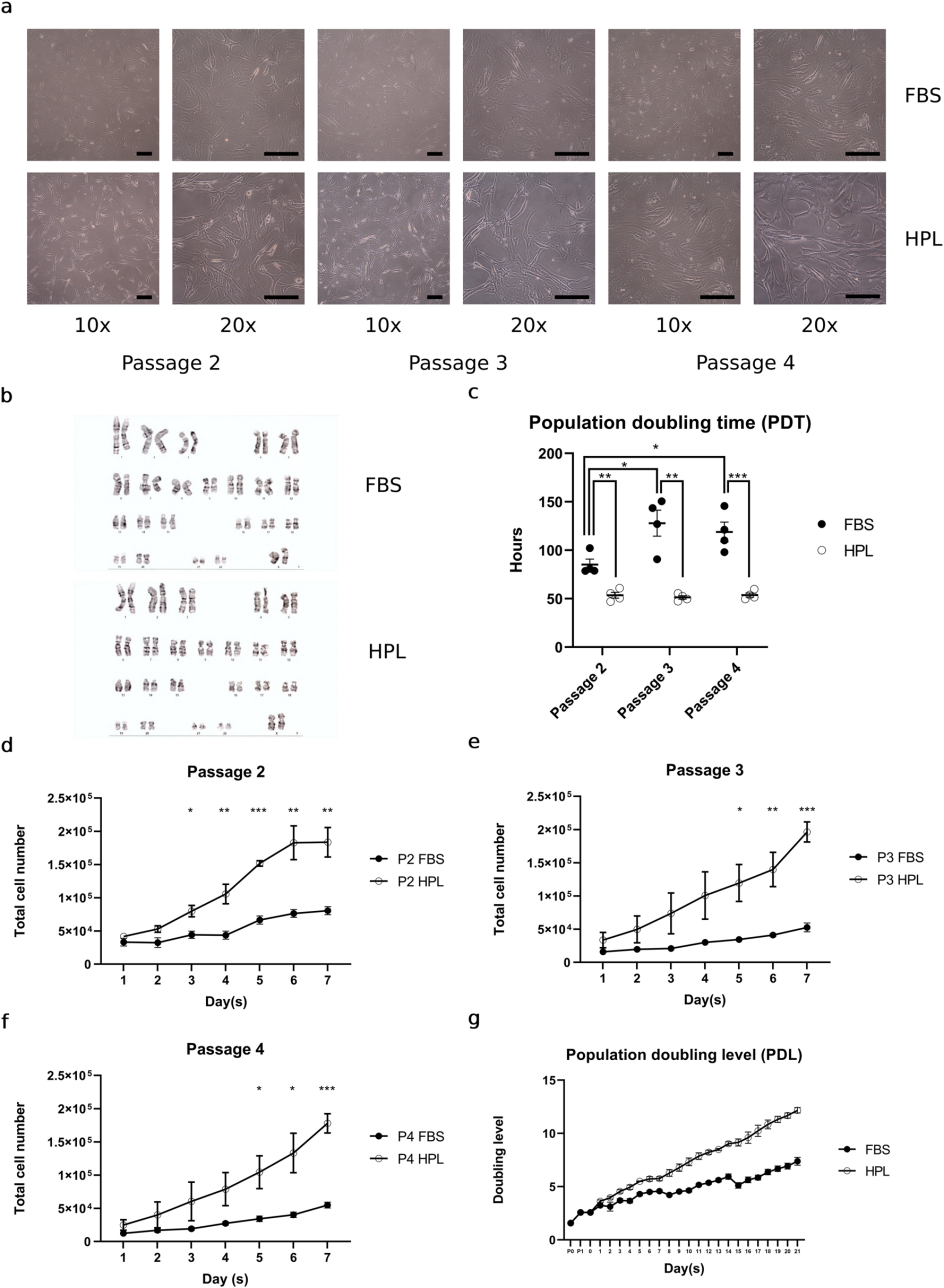
HPL growth supportive properties for HACs expansion. (a) Morphological alteration of FBS-HACs and HPL-HACs. Representative HAC morphology were taken during P2-P4 *in*-*vitro*-expansion by 10 × and 20 × objective magnification, scale bar = 200 μm. (b) Cytogenetic (karyotyping) study ensured normal chromosomal number in both FBS-HACs and HPL-HACs. (**c**) PDT of FBS-HACs and HPL-HACs during P2-P4 *in*-*vitro*-expansion. (**d**, **e**, and **f**) Total cell number at P2-P4. (**g**) Cumulative PDL of HPL-HACs from the consecutive 21 days *in*-*vitro*-expansion. All experiments were performed with *n* = 4, Analyzed data were considered statistical significant at *p* < 0.05 (*), *p* < 0.01 (**) and *p* < 0.001 (***).

In this study, we compared the effects of HPL (HPL-HACs) to those of a conventional HAC growth medium condition (FBS-HACs), during passage 2 (P2) to passage 4 (P4) in vitro HAC expansion. FBS-HACs progressively expanded to a broad morphology on a plastic cell-culture surface. In turn, HPL-HACs became elongated and adopted a spindle cell shape with superior growth-assisted characteristics compared with FBS-HACs. HPL-HACs maintained their morphology (Fig. 1a) and exhibited a normal chromosomal number (Fig. 1b) during P2-P4 in vitro expansion. HPL-HACs significantly decreased PDT and maintained its low PDT level from P2 to P4 as 53.6 ± 3.0, 51.4 ± 1.8, and 53.8 ± 2.2 h at P2 (*p* = 0.003), P3 (*p* = 0.001), and P4 (*p* < 0.001), respectively, compared with FBS-HACs. Meanwhile, FBS-HACs increased its PDT from 85.2 ± 5.7 h at P2, which was significantly increased to 128.0 ± 13.4 h and 118.9 ± 10.2 h at P3 (*p* = 0.026) and P4 (*p *= 0.028), respectively (Fig. 1c). HPL-HACs proliferation capacity was significantly enhanced by increasing of total cell count at P2-P4 (Fig. 1d, e, and f). HPL-HACs progressively increased cumulative population doubling level (PDL) and estimated to increase total HAC expansion for approximately 27.5-fold compared with FBS-HACs within 21 days *in-vitro*-expansion period.

### HPL reduced cellular senescence and upregulated chondrogenic markers during passage 2 (P2) to passage 4 (P4) in vitro HAC expansion

**Figure 2:**
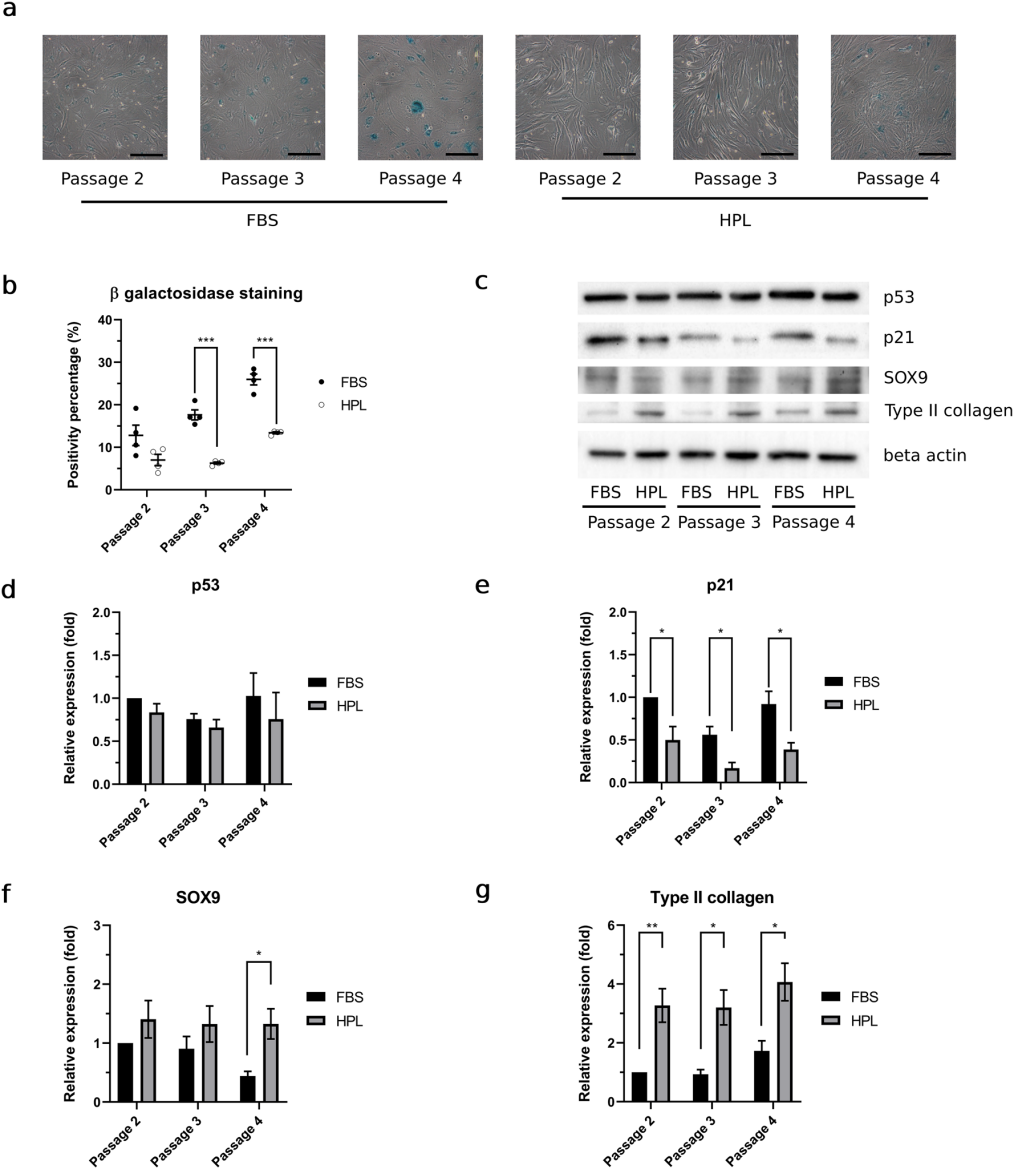
HPL reduced cellular senescence while maintain stable and upregulated chondrogenic marker expression in HACs. (**a**) Representative of HACs senescence staining were taken with 20 × objective lens magnification, scale bar = 200 μm. (**b**) Percentage of senescence staining positivity in FBS-HACs and HPL-HACs. (**c**) Representative western blot bands of p21, p53, SOX9, type II collagen, and β-actin of FBS-HACSs and HPL-HACs. Trend of expression throughout P2-P4 of (**d**) p53, (**e**) p21, (**f**) SOX9, and (**g**) type II collagen. All experiments were performed with *n* = 4, Analyzed data were considered statistical significant at *p* < 0.05 (*), *p* < 0.01 (**) and *p* < 0.001 (***).

Under microscopic evaluation, the senescence percentage of FBS-HACs and HPL-HACs was progressively increased by each passage. However, HPL-HACs exhibited a lower senescence percentage compared to FBS-HACs. Representative microscopic HAC images was shown in Fig. 2a. Compared to FBS-HACs, HPL-HACs senescence percentage was slightly decreased at P2; however, a significant decrease in this parameter was observed in P3 (*p* < 0.001) and P4 (*p* < 0.001) (Fig. 2b). In addition to senescence staining, we examined the expression of the p53 and p21 proteins in P2-P4 HACs; representative bands from western blot analyses are shown in Fig. 2c. There was no significant increasing or decreasing trend in p53 and p21 expression in both FBS and HPL conditions, compared with P2 FBS-HACs, during P2-P4 expansion (Fig. 2d and e). Significant downregulating effects in HPL-HACs were solely observed for p21 expression at P2 (*p* = 0.020), P3 (*p* = 0.015), and P4 (*p* = 0.020) (Fig. 2e).

With high relevance for cellular senescence, we examined the expression of HAC chondrogenic markers, including SOX9 and type II collagen, using western blot analysis. FBS-HACs progressively exhibited a downregulation tendency of SOX9 expression (Fig. 2f). In contrast, SOX9 and type II collagen were upregulated in HPL-HACs condition (Fig. 2f and g). Stabilized and upregulated SOX9 in HPL-HACs, leading to the significant and stable upregulation of type II collagen expression (Fig. 2g).

### HPL enhancement of HAC proliferation capacity through the enrichment of bone morphogenetic protein 2 (BMP-2)–TAK1–p38 signaling transduction disrupted by a potent selective type I bone morphogenetic protein receptor (BMPRI) inhibitor, LDN193189.

**Figure 3:**
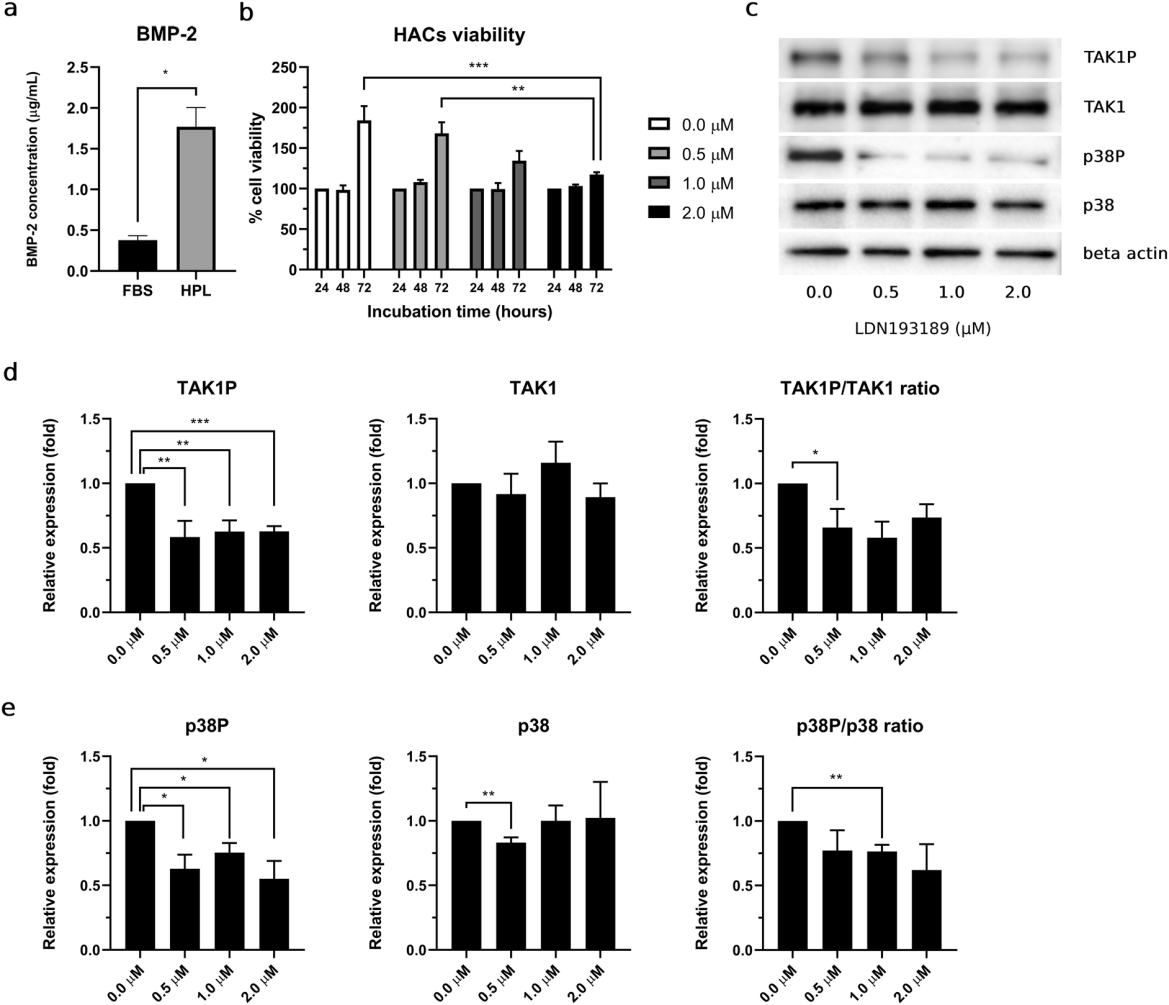
Enrich BMP-2 in HPL enhanced HACs proliferation via TAK1 p38 signaling transduction, and interference of LDN193189 as potent selective type I bone morphogenetic protein receptor (BMPRI) inhibitor. (**a**) Bone morphogenetic protein 2 (BMP-2) concentration in HPL, compared to FBS. (**b**) Percentage HACs viability at 24, 48 and 72h incubation. (**c**) Representative of bands of phosphorylated TAK1 (TAK1P), p38 (p38P), total TAK1 (TAK1), p38 (p38) and beta actin from western blot analysis. Relative expression of phosphorylated, total protein and phosphorylation ratio of (**d**) TAK1 and (**e**) p38. All experiments were performed with *n* = 4, Analyzed data were considered statistical significant at *p* < 0.05 (*), *p* < 0.01 (**) and *p* < 0.001 (***).

In this study, we observed significantly high bone morphogenetic protein 2 (BMP-2) concentration in HPL compared with FBS. BMP-2 concentration in HPL exhibit approximately 4.69-fold as 1.77 ± 0.24 μg/ml for HPL vs. 0.38 ± 0.05 μg/ml for FBS (Fig. 3a). HACs viability in all conditions exhibited similar viability percentage at 24h and 48h LDN193189 incubation period. Compared with 24h, which was employed as the control condition, HACs at 48h exhibited a slightly increased viability percentage. However, at the 72h, HACs in all culture conditions showed an increased viability percentage, by 1.8-, 1.65-, 1.35-, and 1.2-fold in the control and 0.5, 1.0, and 2.0 μM LDN193189, respectively (Fig. 3b).

To validate the successful LDN193189 BMP-2 signaling interferences, the expression of BMP-2-mediated downstream signaling molecules, TAK1 and p38, was studied. Representative bands of total TAK1 and p38, as well as their phosphorylated forms, are shown in Fig. 3c. LDN193189 inhibited phosphorylated TAK1 and p38 expression in HACs. Compared with the control condition, phosphorylated TAK1 (TAK1P) was significantly downregulated in the presence of LDN193189 at 0.5 μM (*p* = 0.016), 1.0 μM (*p* = 0.005), and 2.0 μM (*p* < 0.001). The phosphorylation ratio of TAK1P/TAK1 in HACs was significantly reduced at 1.0 μM (*p* = 0.015) and 2.0 (*p* = 0.042) μM (Fig. 3d). Expression of phosphorylated p38 (p38P) was downregulated by LDN193189. Moreover, significant downregulation of p38P was detected at 0.5 μM (*p* = 0.015), 1.0 μM (*p* = 0.016), and 2.0 μM (*p* = 0.018) LDN193189. The phosphorylation ratio of p38P/p38 in all treatment conditions was decreased, whereas significant downregulation was observed in the 0.5 μM condition (*p* = 0.004) (Fig. 3e).

### HPL anti-senescence properties mediated by BMP-2 signaling transduction decreased and disrupted by the presence of LDN193189

**Figure 4:**
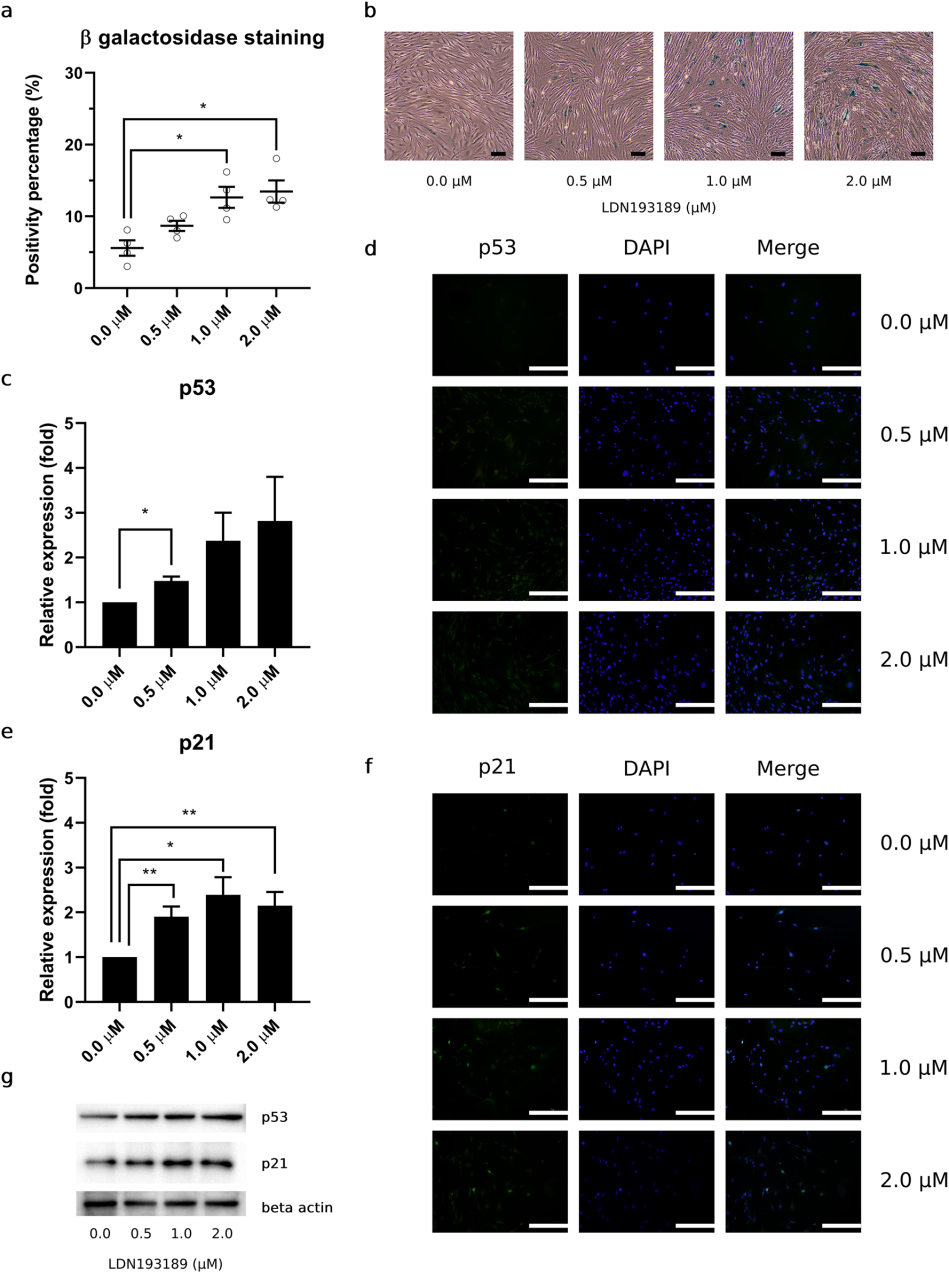
Lowering of HPL BMP-2 signaling transduction leading to increase HACs senescence and p53 p21 upregulation. (**a**) Percentage of HACs senescence from β-galactosidase staining in the present of LDN193189 treatment. (**b**) Representative of HPL-HACs senescence were observed under 20 × objective lens magnification, scale bar = 100 μm. (**c**) Relative expression and (**d**) fluorescence signal of p53 were observed, as similar to (**e**) relative expression and (**f**) fluorescence signal of p21 expression, scale bar = 500 μm. All experiments were performed with n = 4, Analyzed data were considered statistical significant at *p* < 0.05 (*), *p* < 0.01 (**) and *p* < 0.001 (***).

To study the effects of LDN193189 and its HPL BMP-2 signaling interference, we used P4 HPL-HACs to perform this part of experiments. In the presence of LDN193189, P4 HPL-HACs senescence percentage was increased, as observed using senescence-associated β-galactosidase staining assay. In control condition, HPL-HACs, senescence percentage was found as 5.58 ± 1.08%. Progressive increase of cellular senescence percentage was found in treated condition as 8.68 ± 0.71, 12.65 ± 1.46, and 13.47 ± 1.55% in 0.5, 1.0, and 2.0 μM LDN193189 condition, respectively. Significant increase of senescence percentage was detected at 1.0 μM (*p* = 0.008) and 2.0 μM (*p* = 0.006), compared to control condition (Fig. 4a). Representative HAC β-galactosidase staining was assessed and exhibited the same trend toward a progressive increase in blue-color-stained HACs under microscopic observation (Fig. 4b).

In the presence of LDN193189, p53 expression was upregulated in a dose-dependent manner. Significant upregulation was observed in the 0.5 μM LDN193189 condition (p = 0.010) (Fig. 4c). According to western blot results, immunofluorescence staining additionally demonstrated an increased fluorescence signal for cytoplasmic p53 in all LDN193189 treated conditions (Fig. 4d). Apart from p53, we observed upregulated of p21 expression in all LDN193189 treated conditions, significant increase p21 expression was found at 0.5 μM (*p* = 0.008), 1.0 μM (*p*  = 0.012), and 2.0 μM (*p*  = 0.010) LDN193189 condition (Fig. 4e). Similar to p53, immunofluorescence staining of p21 revealed an increased fluorescence signal in all treatment conditions compared with the control. However, unlike p53, the p21 fluorescence signal was not only observed as a cytoplasmic protein but also as having a high intranuclear accumulation in HACs (Fig. 4f). Representative p53, p21, and β-actin bands from western blot analyses are shown in Fig. 4g.

### HPL chondrogenic induction properties disrupted by LDN193189, and reduced BMP-2–TAK1–p38 signaling by LDN193189 directly affected the expression of the HAC chondrogenic markers type II collagen and SOX 9

**Figure 5:**
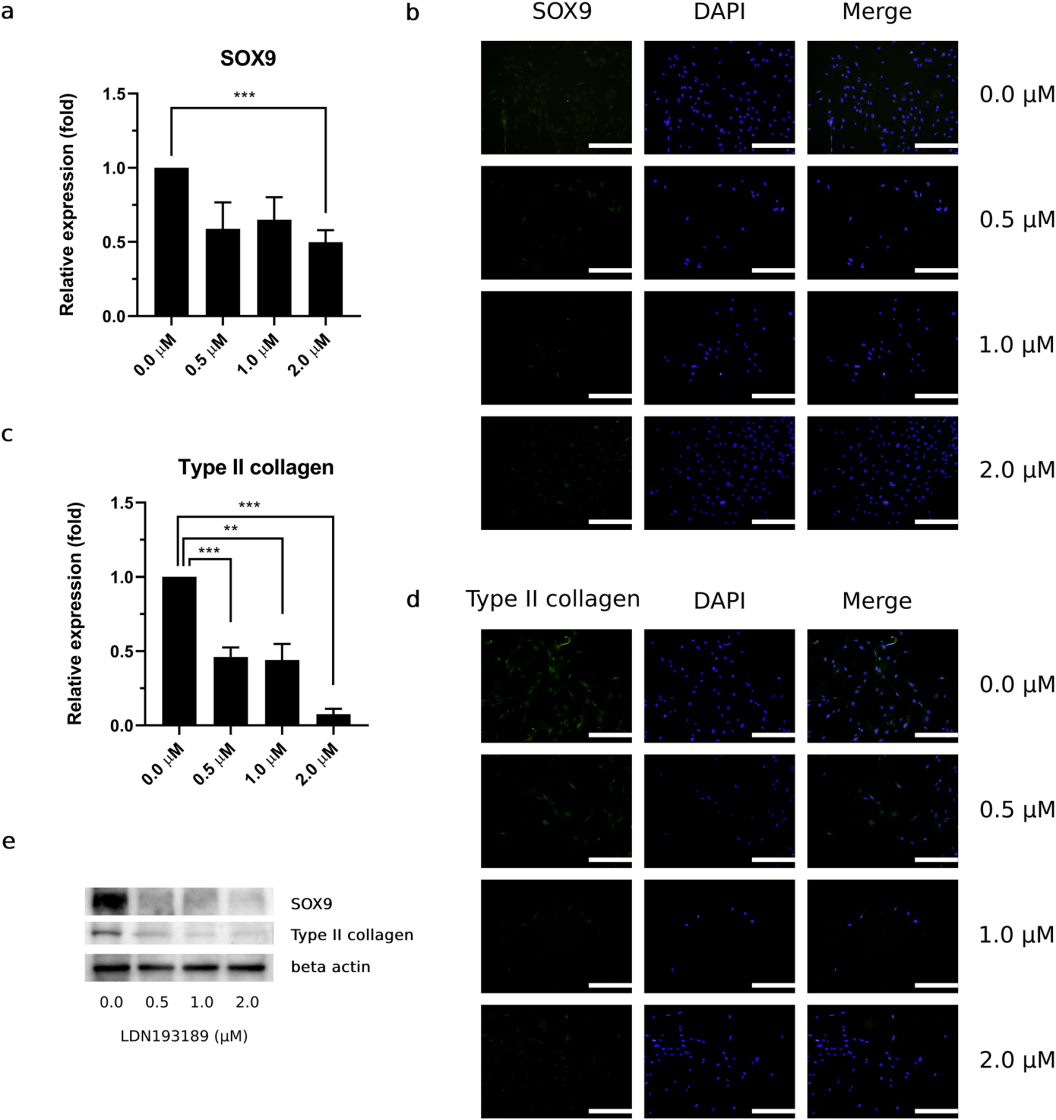
Lowering of BMP-2 TAK1 and p38 expression directly affected type II collagen and SOX9 expression in HACs with increasing LDN193189 concentration. Expression of SOX-9 in HPL-HACs by (**a**) western blot analysis and (**b**) immunofluorescence staining. Relative expression of type II collagen was observed by (**c**) western blot analysis, (**d**) fluorescence signal of type II collagen were observed under fluorescence microscope, scale bar = 500 μm. (**e**) Representative bands of SOX9, type II collagen and β-actin from western blot analysis. All experiments were performed with *n* = 4, Analyzed data were considered statistical significant at *p* < 0.05 (*), *p* < 0.01 (**) and *p* < 0.001 (***).

In this part of study, LDN193189 hindered expression of HAC chondrogenic markers, including SOX9 and type II collagen. SOX9 expression was downregulated in all treatment conditions, significant downregulation was observed at 2.0 μM (*p* < 0.001) (Fig. 5a). Consistent with SOX9 protein expression, SOX9 fluorescence signal was decreased in all LDN193189-treatment conditions (Fig. 5b). Moreover, expression of type II collagen expression was significantly downregulated in a dose-dependent manner. Compared with the control condition, downregulation of type II collagen was observed at 0.5 μM (*p* < 0.001), 1.0 μM (*p* = 0.002), and 2.0 μM (*p* < 0.001) LDN193189 (Fig. 5c). By lowering BMP-2–TAK1–p38 signaling, type II collagen expression was also downregulated by the increasing LDN193189 concentration, as assessed based on the fluorescence signal (Fig. 5d). Representative SOX9, type II collagen, and β-actin bands from western blot analyses are shown in Fig. 5e.

## Discussion

Currently, difficulties in HAC expansion were mentioned as HAC phenotypic changes, including low proliferation capacities, increase cellular senescence, and HAC dedifferentiation. The alteration affected quality of clinical grade HACs, in vitro expansion capacities and therapeutic outcome of the current cartilage-regeneration strategy, i.e., ACI^38^. To improve qualities of clinical grade HACs, alternative medium supplements such as human platelet lysate (HPL), human serum (HS), and serum-free medium are count as promising human derived alternative HACs supplement^39^. From the study of Philippe V, HACs culture with HS and HPL exhibit similar characteristics including cell morphology and nearly identical growth kinetic^40^. Benefits of HS known as an ease of sample preparation and comparable HACs quality for individual patient use. To scale up production, HPL are suitable materials for industrial batch production in order to minimize donor biological variation and maintain the process under current good manufacturing practice (cGMP) production^40^. Alternative human-derived HAC culture medium supplement, HPL, was applied for comparison with the conventional xenogenic supplement, FBS. The main purpose of this study was to elucidate the role of a HPL in HAC expansion, and how HPL affects HAC characteristics, including the cellular senescence and dedifferentiation property.

To observe HAC phenotypic changes, we compared HPL-HACs with FBS-HACs. In our study, FBS-HACs rapidly underwent dedifferentiation, after adhered to the plastic surface. HAC morphology was progressively shifted from a round and tiny shape with a cobble-stone-like morphology at P0 to an expanded cell shape during multiple HAC sub-passaging in FBS-HACs. Morphological shifting indicate the alteration of intracellular molecular networks, leading to cellular senescence and HAC dedifferentiation^41,42^. HACs at passage 4 cultured with either FBS or HPL demonstrated normal chromosomal number and pattern. HPL-HACs exhibited a distinct morphology with a significant increase in proliferative capacities compared with FBS-HACs. HPL afforded superior growth-supportive characteristics for HACs. Moreover, HPL was estimated to increase HPL-HACs expansion capacities by 27.5-fold compared with FBS-HACs.

The lowering of proliferative capacities in FBS-HACs was correlated with a progressively increase in HAC cellular senescence percentage, as well as upregulation of mitotic arrested protein, p21 in FBS-HACs. After P2, HPL exerted its benefits by demonstrated a significant decrease senescence percentage as well as p21 protein expression in HPL-HACs. Moreover, HPL demonstrated the beneficial effects to maintain the upregulation level of chondrogenic markers including type II collagen and SOX9 during in vitro HAC expansion. Furthermore, we observed and found that the ratio of *Col2A1* and *Col1A1* mRNA expression, which reflect a ratio of hyaline cartilage to fibrocartilage regeneration, was upregulated in P2 to P4 HPL HACs, as shown in supplementary figures, which overcome the gradually dedifferentiated FBS-HACs. However, further study in the aspect of HPL and chondrocyte hypertrophy still needed (Supplementary Fig. 7).

HPL-induced growth-assistive effects might occur via the multiple essential growth factors present in HPL such as TGFβ1, TGFβ3, FGF2, BMP-2, and BMP-7^43–47^. From the study of Zhang Y et al., enriched TGFβ1 and FGF2 in platelet rich plasma (PRP) lysate was reported to enhance chondrocyte proliferation and provide new biological activity for PRP as the initiator of joint repairment in clinical application, compared to serum free medium^46^. Toward an advancement of in vitro HACs expansion, TGFβ1 and FGF-2 have been studied as promising HACs growth inducers^48^. Moreover, BMP-2 synergistic effect with FGF-2 and TGFβ1 was found as HACs proliferation and glycosaminoglycan aggregate inducer^49,50^. In our study, a significantly high BMP-2 content was detected in HPL and might involve growth assistive characteristics and type II collagen synthesis inducer as described by Sekiya et al.^51^. However, our study investigated high concentration of BMP-2 with approximately 1.77 ± 0.24 μg/ml for HPL and 0.38 ± 0.05 μg/ml for FBS. The variation in BMP-2 concentration would be from the technique of measurement, indirect ELISA technique. In-house produced HPL, which suspended in plasma, was used for direct coating in ELISA reaction chamber. Coating with plasma samples affected the signal detection phase, such as cross-reactivity of antibodies and excess substances due to incomplete washing^52^. Nowadays, detection for BMP-2 and other growth factors is widely studied using sandwich ELISA technique^46,53^. To investigate the role of HPL-BMP-2 through its receptor, we employed a selective BMPRI inhibitor, LDN193189, which successfully interfered HPL-BMP-2 signaling transduction through the significant downregulation of phosphorylated TAK1 (TAK1P) and p38 (p38P) expression.

Beneficial characteristics of HPL, including HAC proliferation enhancement, anti-senescence, and chondrogenic induction, were involved with HPL-BMP-2 and its BMPRI signaling transduction. Our results indicated the vital role of the HAC proliferation enhancement via BMP-2–TAK1–p38 signal transduction. In addition, HPL-BMP-2 involved with a decreasing of the cellular senescence as well as expression of the mitotic arrested protein, p53 and p21. To improve understanding of mechanisms underlying the HPL-BMP-2 anti-senescence, other senescence-associated signaling molecules; including p16 and telomerase activity, might provide interesting information^54,55^. HPL-BMP-2 signaling involved with an increasing of HAC chondrogenic markers expression. Several signaling are reported to be involved in SOX9 expression, such as TGF, Wnt, and Notch signaling transduction; however, the BMP-2 signaling pathways were the first to be identified as directly affecting SOX9 expression^56^. The increasing in LDN193189 concentration led to the downregulation of SOX9; subsequently, unable to maintain the high expression level of type II collagen.

Our study demonstrated the benefits of HPL as an alternative human-derived medium supplement for in vitro HAC expansion. HPL maintained adequate HAC characteristics, thus, overcoming the current limitations of conventional in vitro HAC expansion. Moreover, our findings indicated BMP-2–TAK1–p38 BMP signaling involvement as one of the mechanisms underlying the beneficial effects of HPL. To consolidate and amplify the knowledge of this phenomenon, other signaling pathways, such as TGF-β signaling, the canonical pathway of BMP-2 signaling, and the cross signaling transduction of BMP-4, BMP-6, BMP-7, BMP-9, and BMP-10 in which can be interfered by LDN193189, should be studied further to close the gaps among the complex signaling networks at work. An understanding of the underlying networks and beneficial aspects of HPL will benefit future in vitro HAC expansion strategies, to overcome their current limitations and maximize the qualities of the cellular materials and the cartilage-regeneration efficacy.

## Materials and methods

### HPL preparation

Outdated pooled LPPCs were collected as leftover specimens from the blood bank. The study protocol was approved by the Mahidol University Central Institutional Review Board (COE.MU-CIRB 2020/107.2508) and Nakhon Pathom Provincial Health Office Human Ethic Committee (COA.NPH-REC 048/2020). Briefly, outdated LPPCs were transferred to the laboratory and processed into an HPL via repeated freeze–thaw cycles. Fibrin clots were activated by calcium chloride (Merck, Germany) and removed using heavy-spin centrifugation (Hettich, Germany). The supernatants were collected and filtered through a 0.22-μm sterile syringe filter membrane (Sartorius, Germany) before using them as supplementation as a HAC culture medium.

### Human articular chondrocyte isolation and culture

Human articular cartilage tissues were collected as leftover specimens from patients undergoing total knee arthroplasty at Ramathibodi Hospital, Mahidol University, Bangkok, Thailand. All patients read the donor informed consent forms before specimen collection as part of the study protocol, which was approved by the Human Research Ethics Committee, Faculty of Medicine Ramathibodi Hospital, Mahidol University (COA.MURA2018/987). Articular cartilage tissues were transferred to the laboratory, cleaned, and cut into small pieces prior to digestion using type II collagenase (Worthington, USA). The enzymatic reaction was halted by the addition of Dulbecco’s Modified Eagle’s Medium (DMEM-HG) (Gibco, USA). The digested tissues and cellular components were seeded into plastic cell-culture flasks (Corning, USA). HACs were maintained in a humidified 5% CO_2_ incubator at 37 °C (Thermo Scientific, USA). The medium was changed twice a week. All experiments were performed using cartilage samples from 4 patients in order to observe and analyze patient biological variation (*n* = 4).

### LDN193189 treatment

At passage 4, HACs in the HPL condition were seeded onto a plastic cell-culture surface (Corning, USA) and left overnight, to provide an adhesion period, in a humidified 5% CO_2_ incubator at 37 °C (Thermo Scientific, USA). On the next day, all HPL media were discarded and replaced with freshly prepared 0.0 μM (control condition) and 0.5, 1.0, and 2.0 μM LDN193189 (STEMCELL Technologies, Singapore) in HPL-supplemented medium. All treatment groups (including the control) were incubated in LDN193189 for 72 h before harvesting for experimentation.

### Cytogenetic study, karyotyping

We observed HACs chromosomal pattern and number in passage 4 FBS-HACs and HPL-HACs by using conventional cytogenetic method, G banding technique. In brief, we added colchicine (Sigma-Aldrich, USA) to P4 HACs cultures in order to arrest its mitotic activity. HACs were detached from culture surface, and subsequently treated in hypotonic solution (Merck, Germany) followed by fixation (Merck, Germany). Harvested HACs were dropped onto microscope slides to obtain HACs metaphase spread. G-banded chromosomes were prepared by trypsin treatment (Invitrogen, USA), thereafter, stained in Giemsa solution (Merck, Germany). Chromosome analysis and karyotyping were performed according to standard cytogenetic procedure.

### Total cell counting and population doubling time (PDT) calculation

Human articular chondrocytes were seeded at a density of 5.5 × 10^4^ cells/cm^2^; subsequently, HACs were dissociated and detached from the culture surface using a 0.025% Trypsin EDTA solution (Gibco, USA). Cell counting was performed using hemacytometer (Marienfeld, Germany). The PDT and population doubling level (PDL) were computed by a “cell calculator” tool (doubling-time.com; Roth V., 2006) (roosterbio, 2021) as follows:

$${\text{PDT}} = \frac{{\left( {{\text{Culture}}\;{\text{time}}\;\left( {{\text{hours}}} \right)} \right) \times \log_{10} 2}}{{\left( {\log_{10} \;{\text{harvested}}\;{\text{cell}}\;{\text{number}}} \right) - \left( {\log_{10} {\text{initial}}\;{\text{cell}}\;{\text{number}}} \right)}}$$

$${\text{PDL}} = {\text{PDL}}_{0} + \left( {3.322 \times \log_{10} \frac{{{\text{Harvested}}\;{\text{cell}}\;{\text{number}}}}{{{\text{Initial}}\;{\text{cell}}\;{\text{number}}}}} \right)$$

### Western blot analysis

Lysate samples from HACs were collected in RIPA lysis buffer (Merck, Germany) containing protease inhibitor (EMD Millipore, USA) and phosphatase inhibitor (Roche, Switzerland) cocktails. Protein concentrations were measured using the Pierce® BCA Protein Assay Kit (Thermo Scientific, USA). Relative protein expression was studied by western blot analysis. Briefly, proteins were separated according to molecular weight using sodium dodecyl sulfate–polyacrylamide gel electrophoresis (SDS–PAGE) (Bio-Rad Mini PROTEAN® Tetra Cell System; Bio-Rad, USA) and subsequently blotted onto a polyvinylidene difluoride (PVDF) membrane (Merck, Germany). Unbound areas were blocked by BlockPRO™ (Visual protein, Taiwan) before incubation with a primary antibody against the target protein overnight. The excess antibody was washed off, and the membrane was incubated with the secondary antibody. Protein bands were developed and their intensities were detected by Azure ECL (Atlantis bioscience, Singapore) and the Image Lab™ software Version 4.0 (Bio-Rad, UK), respectively. The primary and secondary antibodies used here are listed in Table 1.

**Table 1:** List of antibodies for western blot analysis.

Cat. no	Antibody	Clones	Isotypes
MABC785	Mouse anti-human SOX9 monoclonal antibody (Merck, Germany)	4B7.1	IgG2bκ
MAB8887	Mouse anti-human Collagen type II monoclonal antibody (Merck, Germany)	6B3	IgG1
2524S	Mouse anti-human p53 monoclonal antibody (Cell Signaling Technology, USA)	1C12	IgG1
2947S	Rabbit anti-human p21 Waf1/Cip1 monoclonal antibody (Cell Signaling Technology, USA)	12D1	IgG
Ab109404	Rabbit anti-human TAK1 (S439) monoclonal antibody (Abcam, UK)	EPR2863	IgG
Ab109526	Rabbit anti-human TAK1 monoclonal antibody (Abcam, UK)	EPR5984	IgG
Ab45381	Mouse anti-human p38 (T180 + Y182) monoclonal antibody (Abcam, UK)	M139	IgG1
Ab170099	Rabbit anti-human p38 monoclonal antibody (Abcam, UK)	E229	IgG
MAB1501	Mouse anti-actin monoclonal antibody (Merck, Germany)	C4	IgG2bκ
7076S	Horse anti-mouse IgG, HRP linked antibody (Cell Signaling Technology, USA)		
Ab205718	Goat anti-rabbit IgG, HRP linked antibody (Abcam, UK)		

### Real time Quantitative reverse transcription polymerase chain reaction (qRT-PCR)

mRNA sample from HACs were collected in Trizol reagent (Invitrogen, USA) at room temperature and extracted with Direct-zol RNA Miniprep Kits (Zymo Research, USA). Extracted mRNA were checked for concentration and purity by NanoDrop 2000 spectrophotometer (Thermo Scientific, USA) and subsequently convert into complimentary (cDNA) sample by using iScript cDNA Synthesis Kit (Bio-rad, USA). Relative mRNA expression of *Col1A1, Col2A1, Col10A1* were calculated and normalized with Ct of *GAPDH* as housekeeping gene. Data were analyzed by CFX Manager™ Software version 3.1 (Bio-Rad, USA) CFX96 Real Time PCR Machine (Bio-Rad, USA).

### Senescence-associated β-galactosidase enzymatic staining

HACs were seeded at a density of 5.5 × 10^4^ cells/cm^2^. The HAC medium was discarded and the cells were rinsed with phosphate-buffered saline (PBS). HACs were fixed and stained with a senescence β-galactosidase staining kit (Cell Signaling Technology, USA) at 37 °C in a dry (no CO_2_) incubator overnight. Positive and negative stainings were evaluated under an inverted phase contrast microscope (Olympus, Japan).

### Immunofluorescence staining

HACs were seeded at a density of 5.5 × 10^4^ cells/cm^2^. The HAC medium was discarded and the cells were rinsed with PBS. HACs were fixed and permeabilized with 1% paraformaldehyde (Merck, Germany) and Triton X-100 (Molekula, UK). HACs were incubated with a primary antibody against the target protein overnight. Excess antibody was washed off and the cells were incubated with the secondary antibody. HACs were mounted with Prolong™ Gold antifade reagent containing DAPI (Invitrogen, USA) and visualized under a BX51 fluorescent microscope (Olympus, Japan). The antibodies used in this experiment are listed in Table 2.

**Table 2:** List of antibodies for immunofluorescence staining.

Cat. no	Antibody	Clones	Isotypes
MABC785	Mouse anti-human SOX9 monoclonal antibody (Merck, Germany)	4B7.1	IgG2bκ
MAB8887	Mouse anti-human Collagen type II monoclonal antibody (Merck, Germany)	6B3	IgG1
2524S	Mouse anti-human p53 monoclonal antibody (Cell Signaling Technology, USA)	1C12	IgG1
2947S	Rabbit anti-human p21 Waf1/Cip1 monoclonal antibody (Cell Signaling Technology, USA)	12D1	IgG
F0232	Rabbit anti-mouse IgG, FITC (Dakocytomation, Denmark)		
Ab150077	Goat anti-rabbit IgG, Alexa Fluor 488 (Abcam, UK)		

### MTT assay

HACs at passage 4 (P4) were seeded at a density of 5.5 × 10^4^ cells/cm^2^. HACs were incubated with LDN193189 for 24, 48, and 72 h in a humidified 5% CO_2_ incubator at 37 °C (Thermo Scientific, USA). At each incubation time point, the HAC growth medium was discarded and replaced with the MTT reagent (Invitrogen, USA) before incubation for 4 h. Formazan crystals were solubilized through dimethylsulfoxide (DMSO) addition (AppliChem, Germany). Absorbance was measured at 570 nm and analyzed using the Skanit^tm^ microplate reader and software (Thermo Fisher Scientific, USA).

### Enzyme-linked immunosorbent assay

Samples and standard recombinant human BMP-2 (Sigma-Aldrich, Germany) were coated at 4 °C overnight. Excess samples were discarded and washed with PBS-Tween (AppliChem, Germany). Unbound areas were blocked with 1% BSA (Invitrogen, USA), then washed to remove blocking agent excess. Reaction chambers were incubated with a mouse anti-human BMP-2 monoclonal antibody (Merck, Germany), followed by a horse anti-mouse IgG HRP-conjugated antibody (Cell Signaling Technology, USA), as primary and secondary antibodies. Signals were developed by the addition of TMB solution (Merck, Germany) and stopped by 1 N HCL (Merck, Germany). Signal absorbance was measured at 450 nm on a Skanit^tm^ microplate reader and software (Thermo Fisher Scientific, USA). A list of the antibodies used in this experiment is provided in Table 3.

**Table 3:** List of antibodies for Enzyme linked immunosorbent assay (ELISA).

Cat. no	Antibody	Clones	Isotypes
SAB1403609	Mouse anti-human BMP-2 monoclonal antibody	4B12	IgG2aκ
7076S	Horse anti-mouse IgG, HRP linked antibody (Cell Signaling Technology, USA)		

### Statistical analysis

All data were expressed as the mean ± SEM from four independent experiments (*n* = 4). The statistical analysis was performed using the GraphPad Prism software (GraphPad Software, Inc., La Jolla, CA, USA), and *t*-tests were used to compare the differences between groups. Statistical significant was set *p < 0.05, **p < 0.01 and ***p < 0.001.

### Supplementary Information

Supplementary Information.

## Data Availability

The datasets generated during and/or analysed during the current study are available from the corresponding author on reasonable request.
