# Supplementary material for: Application of human platelet lysate in chondrocyte expansion promotes chondrogenic phenotype and slows senescence progression via BMP–TAK1–p38 pathway
Source: Sci Rep. 2023 Nov 30;13:21106. doi: 10.1038/s41598-023-48544-0 (PMC10689743; doi:10.1038/s41598-023-48544-0)
Supplement: Supplementary file 1 — Supplementary Information. [file 41598_2023_48544_MOESM1_ESM.pdf]

Application of human platelet lysate in chondrocyte expansion promotes chondrogenic phenotype and slows senescence progression via BMP–TAK1–p38 pathway

Narong Chitchongyingcharoen[1], Tulyapruek Tawonsawatruk[2], Jitrada Phetfong[1], Wrattya Aroontanee[1], Aungkura Supokawej[1*]

1 Department of Clinical Microscopy, Faculty of Medical Technology, Mahidol University, Nakhon Pathom, Thailand
2 Department of Orthopedics, Faculty of Medicine, Ramathibodi Hospital, Mahidol University, Bangkok, Thailand

Correspondence*

Assoc. Prof. Dr. Aungkura Supokawej,
Department of Clinical Microscopy, Faculty of Medical Technology, Mahidol University,
999 Phutthamonthon Sai 4, Salaya, Phutthamonthon, Nakhon Pathom 73170, Thailand
Tel: +66 2 441 4371-5 Fax: +66 2 441 4380 E-mail: aungkura.jer@mahidol.ac.th

Supplementary data

Human articular chondrocyte isolation, morphological alteration during multiple subpassage, and in vitro expansion

Primary HACs were observed as round and tiny cells with a cobble-stone-like morphology. Isolated primary HACs successfully attached to a plastic cell-culture surface within 72 h after isolation. HACs were primarily cultured in a conventional growth medium supplemented with FBS (FBS-HACs). The morphology of primary (passage 0, P0) HACs shifted into an expanded broad polygonal shape within late P0 and early P1. Morphological alterations progressively occurred over four passages *in vitro* HAC expansion. The majority of FBS-HACs adopted a broad polygonal shape and exhibited decreased proliferative capacities at P2-P4. Representative images of HAC morphological alterations from the initially isolated P0-P4 are shown in supplementary figure 1 (figure S1).

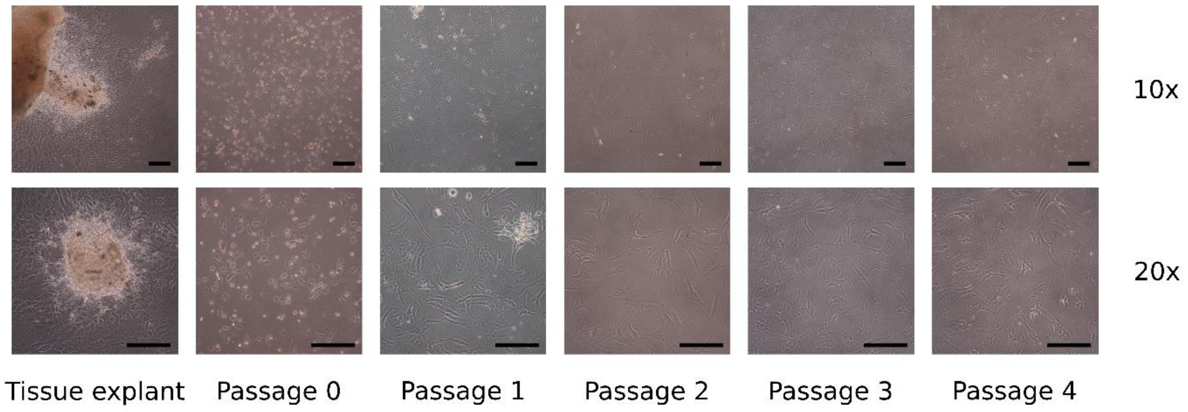

Figure S1. Primary HACs morphology were progressively shifted during *in vitro* expansion. Representative of HACs morphological shift from primary round and tiny morphology at primary passage (P0) into broad shape HACs at P4 were observed by 10x and 20x objective magnification, scale bar = 200 μm.

HPL reduced mitotic arrested protein, p53 and p21, whereas upregulated chondrogenic markers during the four passages of in vitro HAC expansion

(a)

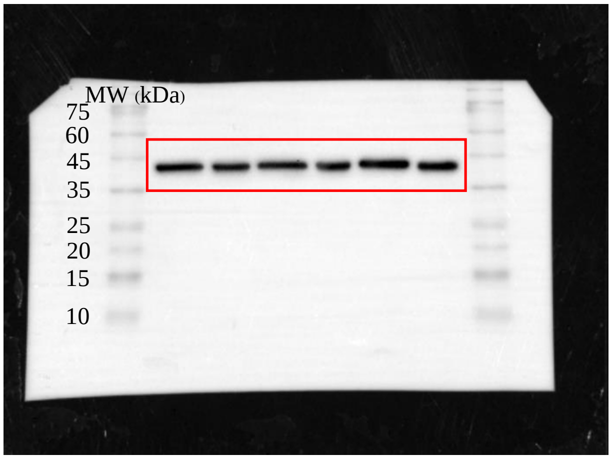

(b)

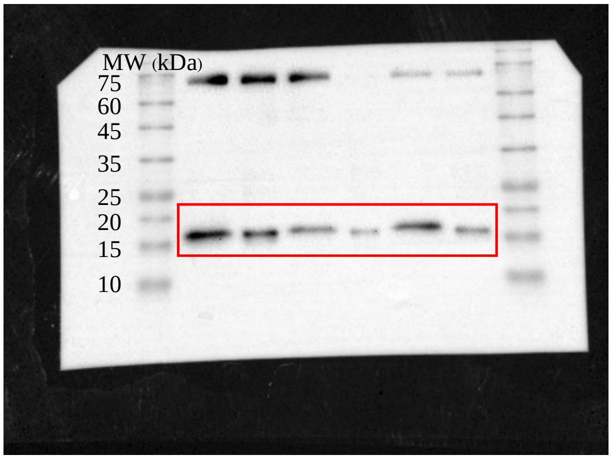

(c)

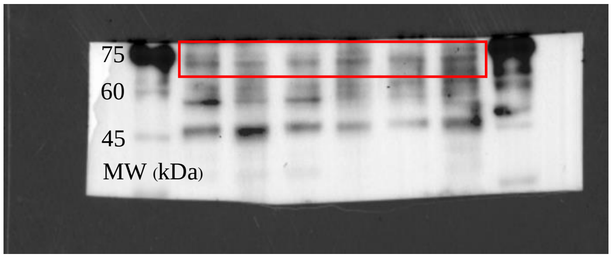

(d)

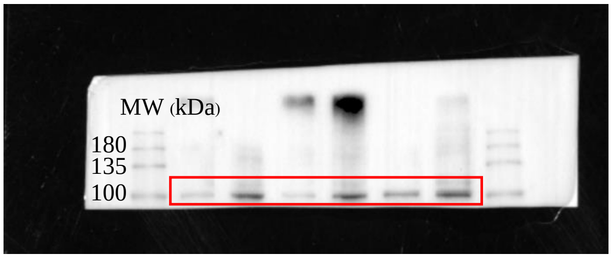

(e)

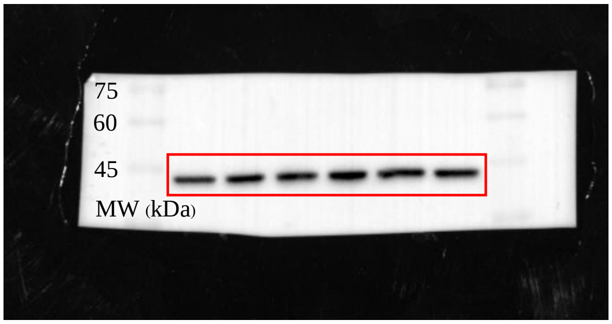

Figure S2. HPL reduced p53 and p21 while maintain stable and upregulated chondrogenic marker expression in HACs. Representative protein bands from western blot analysis were shown as full blot images. The first lane from the left represent P2 FBS-HACs, P2 HPL-HACs, P3 FBS-HACs, P3 HPL-HACs, P4 FBS-HACs, and P4 HPL-HACs. Membranes were stained with the primary antibody to human (a) p53, (b) p21, (c) SOX9, (d) type II collagen, and (d) β-actin. The red squares indicate an area that the representative bands were used in the research article.

Bone morphogenetic protein 2 (BMP-2)–TAK1–p38 signaling transduction disrupted by a potent selective type I bone morphogenetic protein receptor (BMPRI) inhibitor, LDN193189

(a)

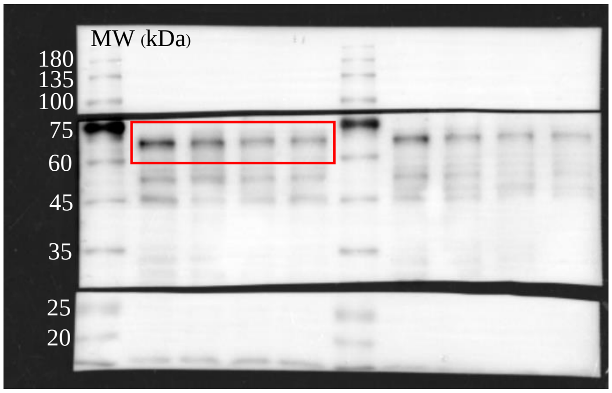

(b)

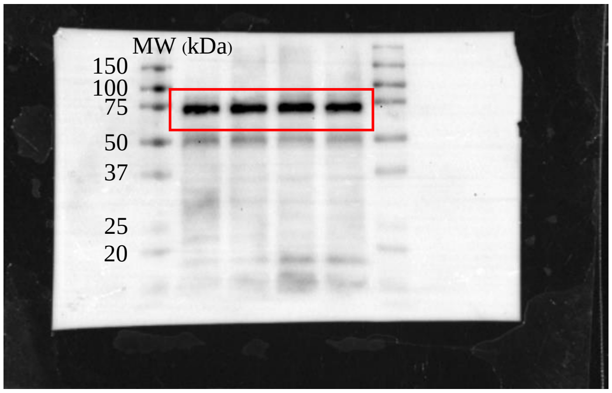

(c)

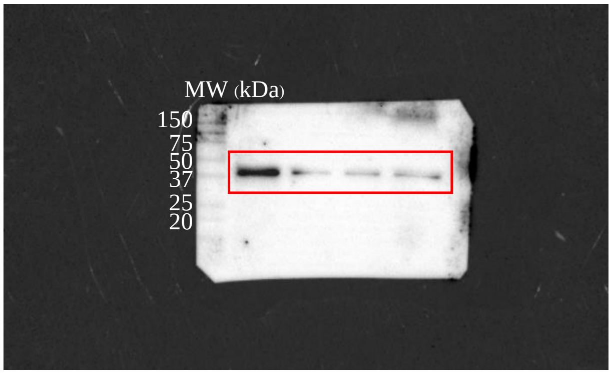

(d)

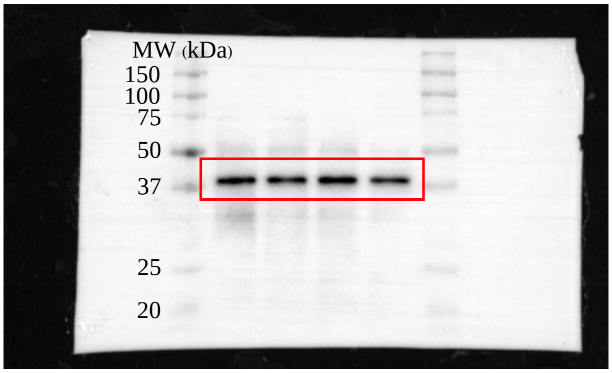

(e)

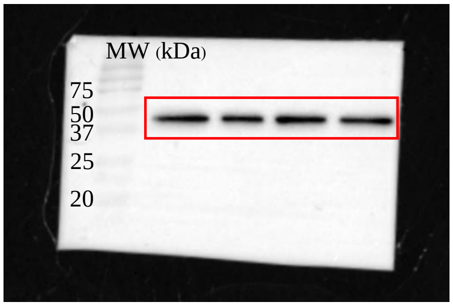

Figure S3. LDN193189 interfered and reduced HPL derived BMP-2-TAK1-p38 signaling transduction. Representative protein bands from western blot analysis were shown as full blot images. The lane represents an increasing of LDN193189 treatment from left to right, as from 0.0 µM (control condition) to, 0.5, 1.0, and 2.0 µM LDN193189 treatment in P4 HPL-HACs. Membranes were stained with the primary antibody to human (a) phosphorylated TAK1, (b) total

TAK1, (c) phosphorylated p38, (d) total p38, and (e) β-actin. The red squares indicate an area that the representative bands were used in the research article.

Lowering of HPL BMP-2 signaling transduction leading to increase HACs senescence and p53 p21 upregulation

(a)

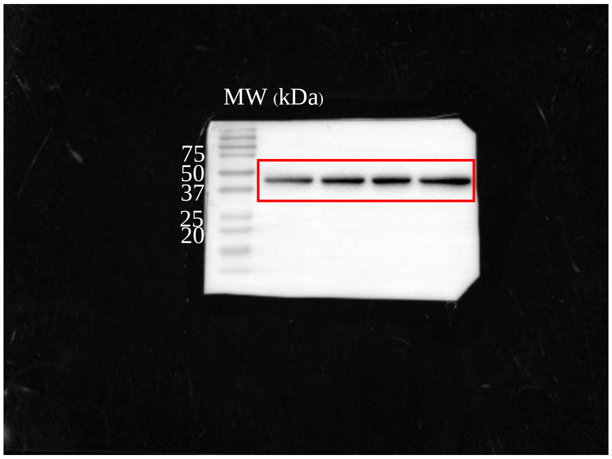

(b)

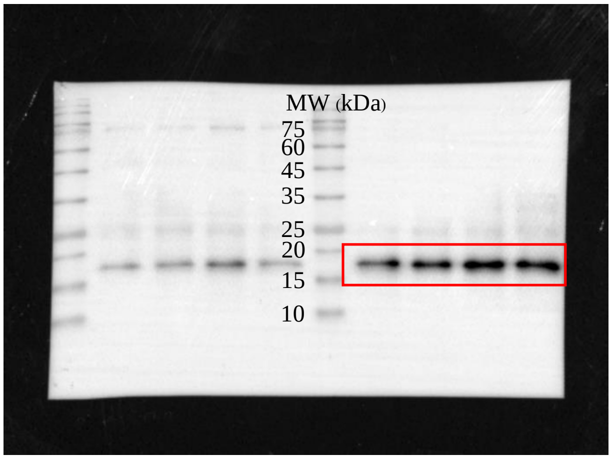

(c)

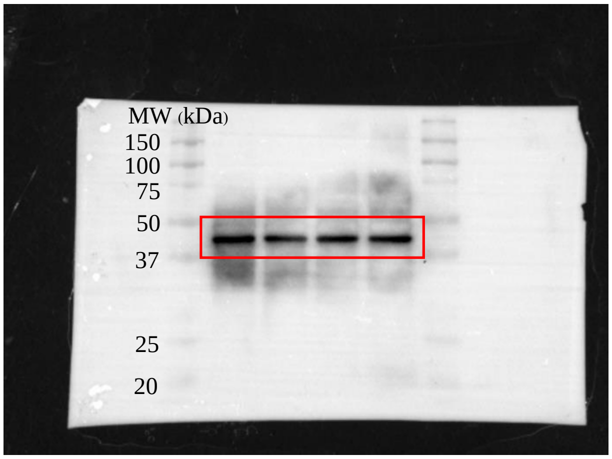

Figure S4. Upregulation of mitotic arrested protein p53, and p21 by LDN193189. Representative protein bands from western blot analysis were shown as full blot images. The lane represents an increasing of LDN193189 treatment from left to right, as from 0.0 µM (control condition) to, 0.5, 1.0, and 2.0 µM LDN193189 treatment in P4 HPL-HACs. Membranes were stained with the primary antibody to human (a) p53 (b) p21 and (c) β-actin. The red squares indicate an area that the representative bands were used in the research article.

Lowering of BMP-2 TAK1 and P38 expression directly affected type II collagen and SOX9 expression in HACs with increasing LDN193189 concentration

(a)

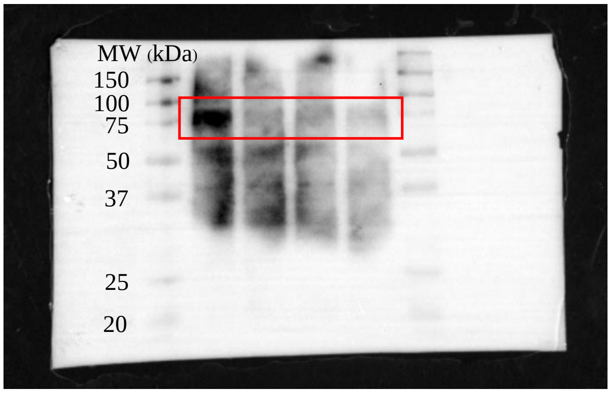

(b)

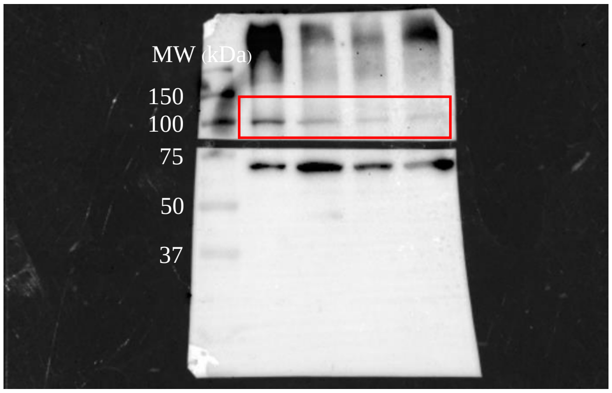

(c)

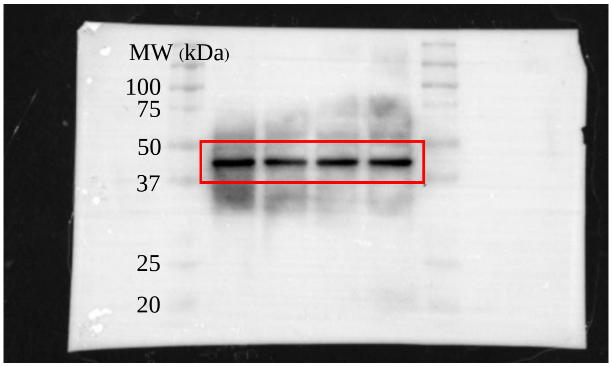

Figure S5. Downregulation of chondrogenic marker expression including; SOX9 and type II collagen by LDN193189. Representative protein bands from western blot analysis were shown as full blot images. The lane represents an increasing of LDN193189 treatment from left to right, as from 0.0 µM (control condition) to, 0.5, 1.0, and 2.0 µM LDN193189 treatment in P4 HPL-HACs. Membranes were stained with the primary antibody to human (a) SOX9 (b) type II collagen and (c) β-actin. The red squares indicate an area that the representative bands were used in the research article.

Raw data set of human bone morphogenetic protein 2 (BMP-2) measurement in HPL and FBS by indirect enzyme linked immunosorbent assay (ELISA)

Table S1. Absorbance data set of HPL, FBS, and BMP-2 standard at 450 nm wavelength.

**Sample**		**Absorbance 1**	**Absorbance 2**
**Human platelet lysate (HPL)**	**HPL1**	0.166	0.154
	**HPL2**	0.162	0.146
	**HPL3**	0.196	0.220
	**HPL4**	0.242	0.213
**Fetal bovine serum (FBS)**	**FBS1**	0.096	0.085
	**FBS2**	0.090	0.078
	**FBS3**	0.076	0.076
	**FBS4**	0.073	0.072
**BMP-2 concentration (µg/ml)**	**2.00**	0.207	0.212
	**1.00**	0.122	0.120
	**0.50**	0.087	0.082
	**0.25**	0.073	0.076
**Blank**		0.056	0.057

HPL increase *Col2A1/Col1A1* mRNA expression ratio during passage 2 (P2) to passage 4 (P4) *in vitro* expansion, by upregulation of *Col2A1* and downregulation of *Col1A1*

(a)

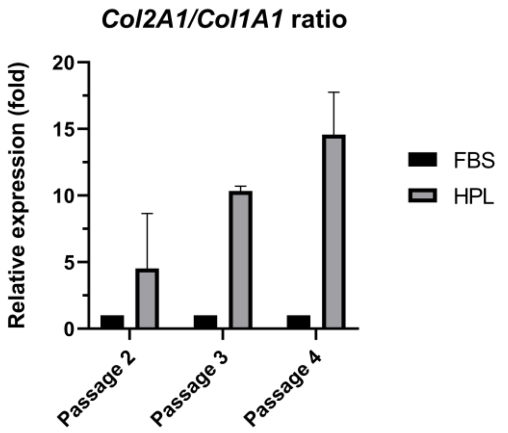

(b)

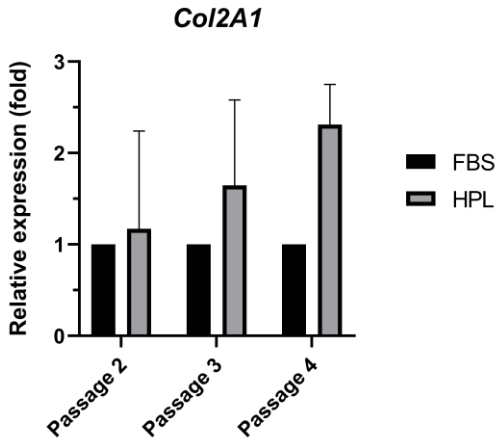

(c)

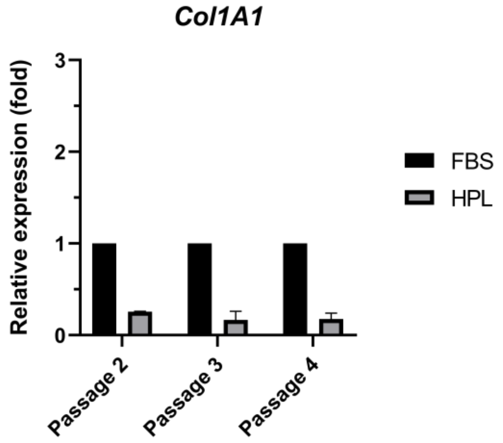

(d)

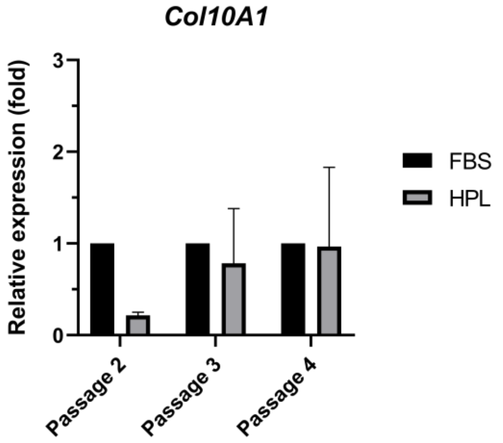

Figure S7 *Col2A1*/*Col1A1* mRNA expression ratio was increased in HPL-HACs. *Col2A1*, *Col1A1*, and *Col10A1* mRNA expression ratio was investigated by real-time RT-PCR. (a) An increase of *Col2A1*/*Col1A1* mRNA expression ratio was observed in P2-P4 HPL-HACs, compared to FBS-HACs. (b) During P2-P4, HPL-HACs exhibit an upregulated *Col2A1*, *(c)* while downregulated *Col1A1* mRNA expression, compared to FBS-HACs. (d) *Col10A1* expression of

HPL-HACs is downregulated, compared to FBS-HACs. Data were shown as mean values from 2 donor samples (n=2).

Table S2. Lists of forward and reverse primers for real-time RT-PCR analysis

**Gene**	**Primer type**	**Primer sequences (5' to 3')**
Col1A1	FW	GGGCAAGACAGTGATTGAATACA
	RV	GGATGGAGGGAGTTTACAGGAA
Col2A1	FW	CCTGGTCTTGGTGGAAACTT
	RV	CAGAGACACCAGGTTCACCA
Col10A1	FW	GTAAAGGTATAGCAGTAAGAGGAGAGC
	RV	ACTTCCGTAGCCTGGTTTTC
GAPDH	FW	CAACTACATGGTTTACATGTTCCAA
	RV	CAGCCTTCTCCATGGTGGT
